# Inflammatory priming with IL-1β promotes the immunomodulatory behavior of adipose derived stem cells

**DOI:** 10.3389/fbioe.2022.1000879

**Published:** 2022-10-20

**Authors:** Alessandra Colombini, Francesca Libonati, Davide Cangelosi, Silvia Lopa, Paola De Luca, Domenico Antonio Coviello, Matteo Moretti, Laura de Girolamo

**Affiliations:** ^1^ Orthopaedic Biotechnology Lab, IRCCS Istituto Ortopedico Galeazzi, Milan, Italy; ^2^ Unità di Bioinformatica Clinica, IRCCS Istituto Giannina Gaslini, Genoa, Italy; ^3^ Cell and Tissue Engineering Laboratory, IRCCS Istituto Ortopedico Galeazzi, Milan, Italy; ^4^ Laboratorio di Genetica Umana, IRCCS Istituto Giannina Gaslini, Genoa, Italy; ^5^ Regenerative Medicine Technologies Lab, Ente Ospedaliero Cantonale, Laboratories for Translational Research (LRT), Bellinzona, Switzerland; ^6^ Department of Surgery, Ente Ospedaliero Cantonale, Service of Orthopaedics and Traumatology, Lugano, Switzerland; ^7^ Faculty of Biomedical Sciences, Euler Institute, Lugano, Switzerland

**Keywords:** adipose derived stem cells, early osteoarthritis, interleukin 1 beta, inflammatory priming, immunomodulation

## Abstract

Inflammatory processes contribute to osteoarthritis (OA) severity and progression. Mesenchymal stem cells, particularly those derived from adipose tissue (ASCs), are able to sense and control the inflammatory environment. This immunomodulatory potential can be boosted by different priming strategies based on inflammatory stimulation. The aim of the present study is to investigate the transcriptional modulation of a huge panel of genes and functionally verify the predicted immunomodulatory ability of ASCs after interleukin one beta (IL-1β) priming. ASCs were isolated from adipose tissue obtained from three donors and expanded. After stimulation with 1 ng/ml of IL-1β for 48 h, cells were collected for gene array and functional tests. Pooled cells from three donors were used for RNA extraction and gene array analysis. Gene Ontology (GO) enrichment analysis and Gene Set Enrichment Analysis (GSEA) were performed to assess the involvement of the modulated genes after priming in specific biological processes and pathways. Functional co-culture tests of ASCs with T cells and macrophages were performed to assess the ability of primed ASCs to modulate immune cell phenotype. Among the overall genes analyzed in the gene array, about the 18% were up- or down-regulated in ASCs after IL-1β priming. GO enrichment analysis of up- or down-regulated genes in ASCs after IL-1β priming allowed identifying specific pathways involved in the modulation of inflammation and extracellular matrix remodeling. The main processes enriched according to the GSEA are related to the inflammatory response and cell proliferative processes. Functional tests on immune cells showed that primed and non-primed ASCs induced a decrease in the CD3^+^ T lymphocytes survival rate and an anti-inflammatory macrophage polarization. In conclusion, IL-1β priming represents a tailored strategy to enhance the ability of ASCs to direct macrophages towards an anti-inflammatory phenotype and, consequently, improve the efficacy of ASCs in counteracting the OA inflammatory component.

## Introduction

Osteoarthritis (OA) is a degenerative chronic disease in which pro-inflammatory cytokines play a role in shifting the joint homeostasis towards a catabolic environment (Kapoor et al., 2011). The inflammatory processes involved in OA severity and progression are mainly mediated by soluble factors and infiltrating immune cells. The pathology of OA affects many components of the involved joint, from cartilage to subchondral bone, with clear signs of inflammation in synovial tissue, a battlefield for infiltrated immune cells (Scanzello and Goldring, 2012). The warriors in this battle are represented by macrophages and T cells (de Lange-Brokaar et al., 2012). In particular, pro-inflammatory macrophages (M1) guide OA inflammation, allowing for the progression of the pathology (Krenn et al., 2002). T cells promote the infiltration of M1 macrophages into the synovium (Haseeb and Haqqi, 2013), where they release interleukin one beta (IL-1β) and tumor necrosis factor alpha (TNFα) cytokines (Pessler et al., 2008). Pro-inflammatory cytokines are important determinants in degenerative OA processes; they mainly promote an increase in metalloproteinase (MMP3, MMP9 and MMP13) expression and activity with a consequent triggering of cartilage matrix degradation (Bondeson et al., 2006).

Considering the contribution of inflammation in the OA context, therapies addressing only tissue regeneration, without any immunomodulatory property may be inadequate. The modulation of the cytokine pattern and of the immune infiltrate can indeed be promising tools to mitigate the chronicization of degenerative processes and promote joint homeostasis in OA patients. Among the novel treatments able to sense and control the inflammatory environment, mesenchymal stem cells (MSCs) are key players (Bernardo and Fibbe, 2013). Multipotent mesenchymal stem cells are present in the tissue stroma of all adult organs, at perivascular sites, and play an important role in tissue homeostasis and remodeling (Colombini et al., 2019). In particular, MSCs exert immunomodulatory properties (Yoo et al., 2009; Melief et al., 2013; Mun et al., 2018; Colombini et al., 2019) through the direct contact with immune cells or the secretion of paracrine factors (Leto Barone et al., 2013). The benefits of the immunomodulatory effect of MSCs have been demonstrated in the treatment of graft *versus* host disease in different patient cohorts, including also pediatric patients with post-transplant complications based on deregulated immune effector cells (Müller et al., 2008; Kelly and Rasko, 2021).

Adipose-derived mesenchymal stem cells (ASCs) can be isolated from easily harvestable fat, with high cell yield and good proliferative capacity (Mohamed-Ahmed et al., 2018). ASCs are considered a more powerful suppressor of immune response than MSCs derived from other tissues (Nancarrow-Lei et al., 2017). *In vitro* they have been shown to induce the switching of activated M1-like inflammatory macrophages towards a M2-like phenotype (Manferdini et al., 2017). In addition, the intra-articular injection of ASCs to treat severe OA not only drives an immediate local response, but also induces a systemic long-lasting immune modulation, promoting an anti-inflammatory profile in circulating T and B cells (Pers et al., 2018). These abilities are not normally expressed by ASCs, but can be stimulated by an inflammatory microenvironment (Bernardo and Fibbe, 2013). Based on these premises, different strategies based on inflammatory cytokines, such as TNFα, interferon gamma (IFNγ) and IL-1β, have been used to boost the immunomodulatory potential of ASCs and achieve more specific therapeutic effects (Ren et al., 2008; Li et al., 2012; Dorronsoro et al., 2014; Noronha et al., 2019; Ceccarelli et al., 2020; Ragni et al., 2020). In particular, the stimulation of MSCs with TNFα and IFNγ (Krampera et al., 2006; Ryan et al., 2007; Crisostomo et al., 2008; English, 2013) improves the secretory profile of MSCs in an anti-inflammatory and immunomodulatory sense. In particular, this priming results in the polarization of MSCs towards an anti-inflammatory phenotype, producing soluble factors such as indoleamine 2,3-dioxygenase 1 (IDO), prostaglandin E2 receptor EP3 subtype-like (PGE2), nitric oxide (NO) and hepatocyte growth factor (HGF), as well as in the ability of MSCs to induce the polarization of macrophages in an anti-inflammatory M2 sense (Bernardo and Fibbe, 2013). ASCs have been also shown to respond to priming with OA synovial fluid increasing soluble factors and micro-RNAs embedded in extracellular vesicles (EV-miRNAs), producing signals involved in extracellular matrix organization, immune response, cell migration and chemotaxis, which are correlated to their therapeutic effects when interacting with OA joint environment (Ragni et al., 2021). Furthermore, it has been demonstrated that OA synovial fluids does not impair the proliferation of ASCs and upregulates the expression of genes involved in their immunomodulatory potential through a TNF/NF-κB (nuclear factor kappa-light-chain-enhancer of activated B cells) dependent mechanism, making them more effective in inducing regulatory T cells and inhibiting pro-inflammatory macrophages (Sayegh et al., 2019). Data obtained by our research group showed that ASCs primed with IL-1β exhibit a superior reactivity in comparison to non-primed cells. After priming, ASCs showed an increased release of angiogenic factors, including molecules belonging to the insulin growth factor (IGF), platelet derived growth factor (PDGF), transforming growth factor (TGF) β-2 and β-3 families and of IL-1Ra, a critical anti-inflammatory cytokine (De Luca et al., 2019). Further analyses identified few miRNAs with a key role in the control of genes involved in the Wnt pathway, cartilage homeostasis, cell proliferation and inflammatory response as modulated by ASCs after IL-1β-stimulation (Colombini et al., 2021a). This evidence suggests the enhancing ability of IL-1β priming on ASCs.

The aim of the present study is to further investigate and functionally verify the immunomodulatory ability of ASCs after IL-1β priming. Transcriptional changes of a huge panel of genes will be analyzed, allowing the identification of relevant modulated gene sets. This information will be compared with previous data obtained from the analysis of a panel of released factors after priming (De Luca et al., 2019). Finally, functional tests will be performed to verify the predicted immunomodulatory ability of these cells.

## Materials and methods

### Cell isolation, expansion and characterization

Subcutaneous adipose tissue was collected from hip fat deposit tissue of three osteoarthritic patients (two females aged 53 and 56 years and one male aged 41 years) who had undergone total hip arthroplasty. ASCs were isolated by enzymatic digestion of harvested adipose tissue (37°C, 30 min) using 0.075% w/v type I collagenase (Worthington Biochemical, Lakewood, NJ, United States ) as previously reported (Lopa et al., 2014).

ASCs were cultured in minimum essential medium (αMEM) supplemented with 10% FBS (Lonza), 0.29 mg/mL l-glutamine, 100 U/mL penicillin, 100 μg/ml streptomycin, 10 mM 4-(2-hydroxyethyl) piperazine-1-ethanesulfonic acid (HEPES), 1 mM sodium pyruvate (all reagents from Life Technologies, Carlsbad, CA, United States ), adding 5 ng/ml fibroblast growth factor 2 (FGF-2) (PeproTech, Rocky Hill, NJ, United States ) to preserve the chondrogenic potential (Solchaga et al., 2005; Liu and Wagner, 2012) at 37 °C, 5% CO_2_ and 95% humidity. Cells were characterized by flow cytometry at passage 4 to assess the expression of characteristic MSC cell-surface antigens, including CD44, CD73, CD90, and CD105, and the absence of hematopoietic and endothelial markers, including CD14, CD34 and CD45 (Colombini et al., 2021a). Experiments were performed using cells at passage 3.

### Priming with IL-1β

Cells were stimulated with 1 ng/ml of IL-1β for 48 h (Daheshia and Yao, 2008; Kapoor et al., 2011) and were subsequently collected for gene array and functional tests.

### RNA extraction and quality assessment

Pooled cells from three donors were used for RNA extraction by RNeasy Plus Mini Kit (Qiagen, Duesseldorf, Germany). RNase-Free DNase Set (Qiagen) was used for residual genomic DNA digestion, and the isolated RNA was quantified spectrophotometrically (Nanodrop, Thermo Scientific, Rockford, IL, United States). Agilent RNA ScreenTape System (Agilent Technologies, Santa Clara, CA, United States) was used to evaluate the RNA integrity (Schroeder et al., 2006).

### Gene expression microarray

A huge panel of genes of interest (maximum 3,000 genes, including at least five replicates for each gene) was selected and analyzed through a custom gene expression microarray designed through Agilent Technologies (https://earray.chem.agilent.com/earray/, date of access 12 February 2018) (De Luca et al., 2020a; De Luca et al., 2020b; Colombini et al., 2021b).

Each RNA sample was added with a spike mix (prepared with the One Color RNA Spike-In kit) to assess the correct annealing of 10 optimized positive control transcripts to the complementary probes of the array and to evaluate the auto- and cross-hybridization.

Low Input Quick Amp Labeling Kit one-color was used to label and amplify 100 ng of RNA to obtain cRNA that was then purified using RNeasy Plus Mini Kit (Qiagen, Hilden, Germany). Gene Expression Hybridization Kit was used to hybridize the samples on the microarray slide, which was then washed and scanned with SureScan Microarray Scanner. Unless otherwise specified, all the reagents, instruments and software were purchased from Agilent Technologies (Santa Clara, CA, United States).

Data were extracted by Feature Extraction v.12.0 software (Agilent).

### Bioinformatic analysis

Fold change Fc > 2 or <0.5 in the cells exposed to different treatments were described and considered to be of interest. Analysis was performed by Genespring GX software 14.9.

To gain insight on the biological meaning associated with the up- and down-regulated genes in ASCs after IL-1β priming, Gene Ontology (GO) enrichment analysis was carried out using the Cytoscape BINGO plugin (Maere et al., 2005). Genes were classified according to the function and cellular component GO collections. GO terms with *p* value and FDR lower than 0.05 were considered significantly enriched. The concordant modulation of functionally related genes between the gene expression profiles of inflamed and non-inflamed samples were assessed by gene set enrichment analysis (GSEA) (Subramanian et al., 2005). GSEA was used to assess the enrichment of the gene sets belonging to the Hallmark (H) collection retrieved from the Molecular Signature Database (MSigDB) v7.4 (Liberzon et al., 2015).

When multiple probe sets were associated to a gene symbol, the average expression value of all probe sets was assigned to the gene using the GSEA collapse function. Genes were ranked according to their expression on the basis a metric. Diff_of_Classes was used as metric for ranking genes by their expression. For each gene set in a collection, GSEA calculated an enrichment score (ES) and normalized enrichment score (NES) using gene rank. An empirical permutation test using 1,000 gene set permutations was used to estimate the statistical significance of the NES (NOM p-val). When multiple gene sets were evaluated, GSEA adjusted the estimate of the significance level to account for multiple hypothesis testing. To achieve this, GSEA computed the false discovery rate q-value (FDR q-value), which estimated the probability that the NES represented a false positive finding. We considered gene sets containing between 15 and 250 genes. Gene sets with nominal *p*-values lower than 0.05 and FDR q-values lower than 0.05 are considered significantly enriched.

### Characterization of T cells after co-culture

After isolation by Ficoll, 2×10^5^ human peripheral blood cells (PBMCs) were directly co-cultured in DMEM-based medium with 1×10^5^ ASCs, previously primed or not primed for 48 h with 1 ng/ml of IL-1β. Non co-cultured PBMCs were used as control. After 4 days of co-culture, PBMCs were collected and stained with monoclonal anti-human CD3-APC antibody (Clone UCHT1, Biolegend) for gating lymphocytes. PBMCs were also stained with monoclonal anti-human CD4-PE/Cy7 antibody (Clone RPA-T4, Biolegend) and monoclonal anti-human CD8-PerCP antibody (Clone SK1, Biolegend) to evaluate the ability of ASCs to modify the CD4^+^/CD8^+^ T cells ratio. Cells were analyzed on a Cytoflex flow cytometer acquiring a minimum of 10,000 events.

### Modulation of macrophage phenotype

Monocytes were isolated by Ficoll (GE Healthcare) density gradient separation from buffy coats of healthy donors obtained from the local blood bank, using CD14 magnetic microbeads (MACS, Miltenyi), according to the manufacturer’s instructions.

Monocytes were seeded to have a final density of 0.3 × 10^5^ cells/cm^2^ for the experiments with M1 polarized macrophages and of 2 × 10^5^ cells/cm^2^ for the experiments with M0 un-polarized macrophages. Monocytes were cultured in RPMI 1640 (Sigma-Aldrich) added with 10% heat-inactivated FBS, 100 U/mL penicillin, 100 μg/ml streptomycin, 200 mM glutamine (Thermo Fisher Scientific). Monocytes were differentiated into M0 macrophages by adding 20 ng/ml of macrophage colony-stimulating factor (M-CSF, Peprotech Inc, Rocky Hill, NJ, United States) to the medium. In parallel, after 2 days, ASCs were plated on the polycarbonate membrane of trans-wells (Merck, Darmstadt, Germany) at a density of 0.7×10^5^ cells/trans-well and left 3 days in appropriate expansion medium. At day 5, macrophages were either maintained in the M0 state in the presence of 20 ng/mL M-CSF or polarized into the M1 phenotype adding to the culture medium IFN-γ (100 ng/ml, Peprotech) and LPS (100 ng/ml, Sigma-Aldrich) (Mondadori et al., 2023). In parallel, at day 5, ASCs were primed with 1 ng/ml of IL-1β. At day 7, trans-wells seeded with ASCs were transferred to the macrophage plates for 2 days of co-culture. For the co-culture, a mix 1:1 of RPMI-based medium and DMEM-based medium was used. In this phase, M-CSF and polarizing cytokines were removed from the culture medium.

At day 9, macrophages were washed with phosphate buffered saline (PBS), detached with non-enzymatic cell dissociation buffer (Thermo Fisher, Frankfurt, Germany) and gentle scraping, and centrifuged at 500×g for 5 min to collect them. Macrophages were then suspended in MACS buffer (Miltenyi Biotec) and treated with FcR Blocking Reagent (Miltenyi Biotec) for 10 min at 4°C to block unwanted binding of antibodies to human Fc receptor. Afterwards, cells were stained to evaluate the expression of cell surface markers with the following antibodies: anti-human CD80-APC (Clone REA661, Miltenyi Biotec) for M1 phenotype and anti-human CD206-FITC (Clone 15-2, Biolegend) for M2 phenotype. Unstained cells were used as a negative control. All the stains were performed at 4 °C for 20 min in the dark. Data were acquired using a Cytoflex flow cytometer (Beckman Coulter Brea) acquiring a minimum of 10,000 events.

### Statistical analysis

The normality of data distribution was assessed by Kolmogorov Smirnov test. Unpaired Student’s t test was used to compare control cells and co-cultured cells. The level of significance was set at *p* ≤ 0.05. Statistical analysis was performed using GraphPad software (GraphPad Prism v5.00, La Jolla, CA, United States ).

## Results

### Gene array analysis identified specific biological processes modulated in ASCs by IL-1β priming

To assess the transcriptional changes induced in ASCs after IL-1β priming, we compared the gene expression profile between ASCs with or without IL-1β priming. Among the 2079 genes analyzed in the gene array (De Luca et al., 2020a), 384 (18.4%) were up-regulated (fold change>2) and 372 (17.9%) were down-regulated (fold change<0.5) in ASCs after IL-1β priming (Supplementary Table S1).

**FIGURE 1:**
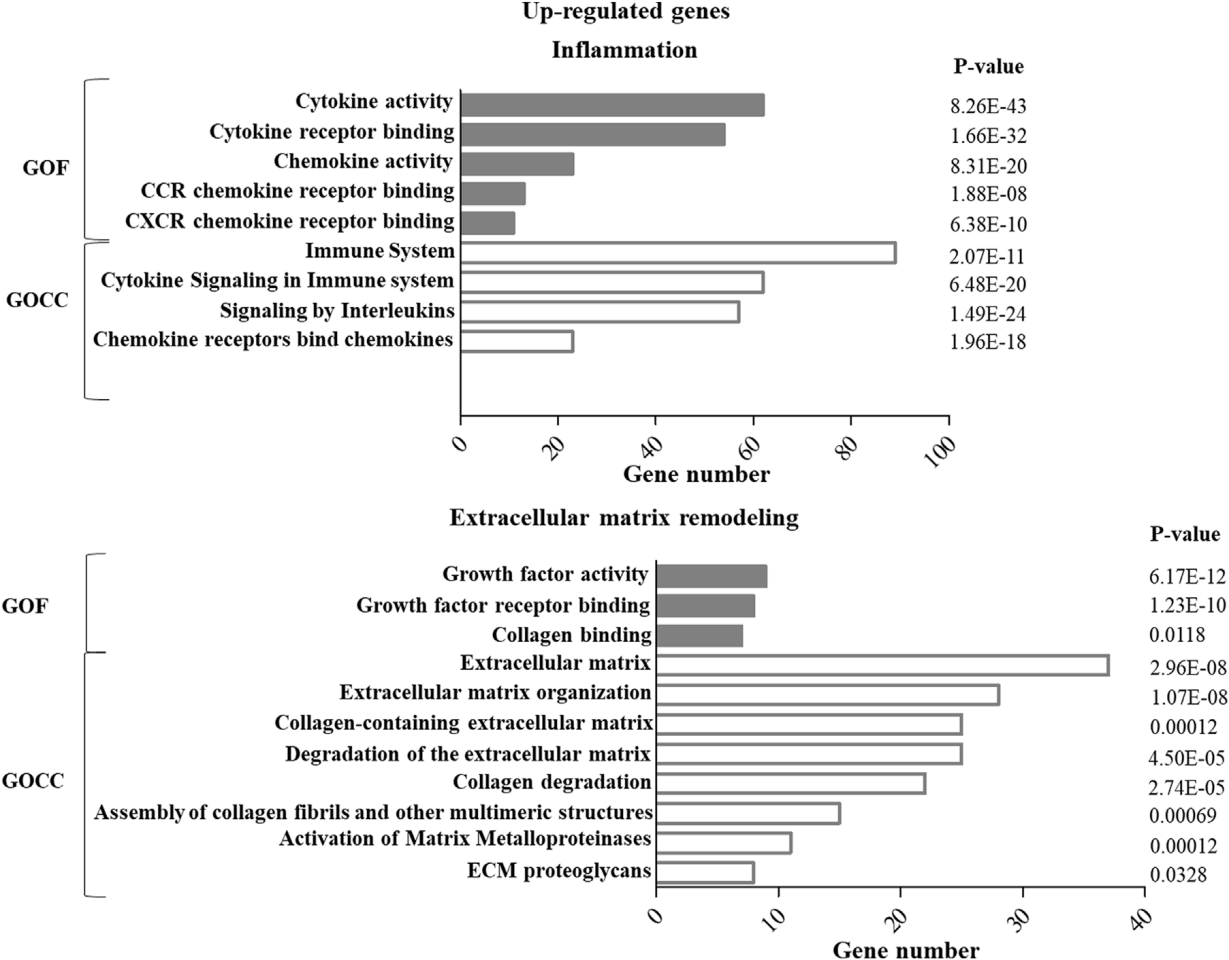
GO enrichment analysis of up-regulated genes in ASCs after IL-1β priming. Up-regulated genes were analyzed using the GO function (GOF) and cellular component (GOCC) collections. A GO term was significantly enriched if *p*-value ≤ 0.05 and FDR ≤0.05. Genes could be comprised in more than one term depending on the function of the encoded protein. The graph shows the most relevant GO terms. The GO term name is reported on the *y*-axis; the number of enriched genes for each term is indicated on the *x*-axis. GO terms are listed by decreasing number of genes. The *p* value for each GO term is indicated.

**FIGURE 2:**
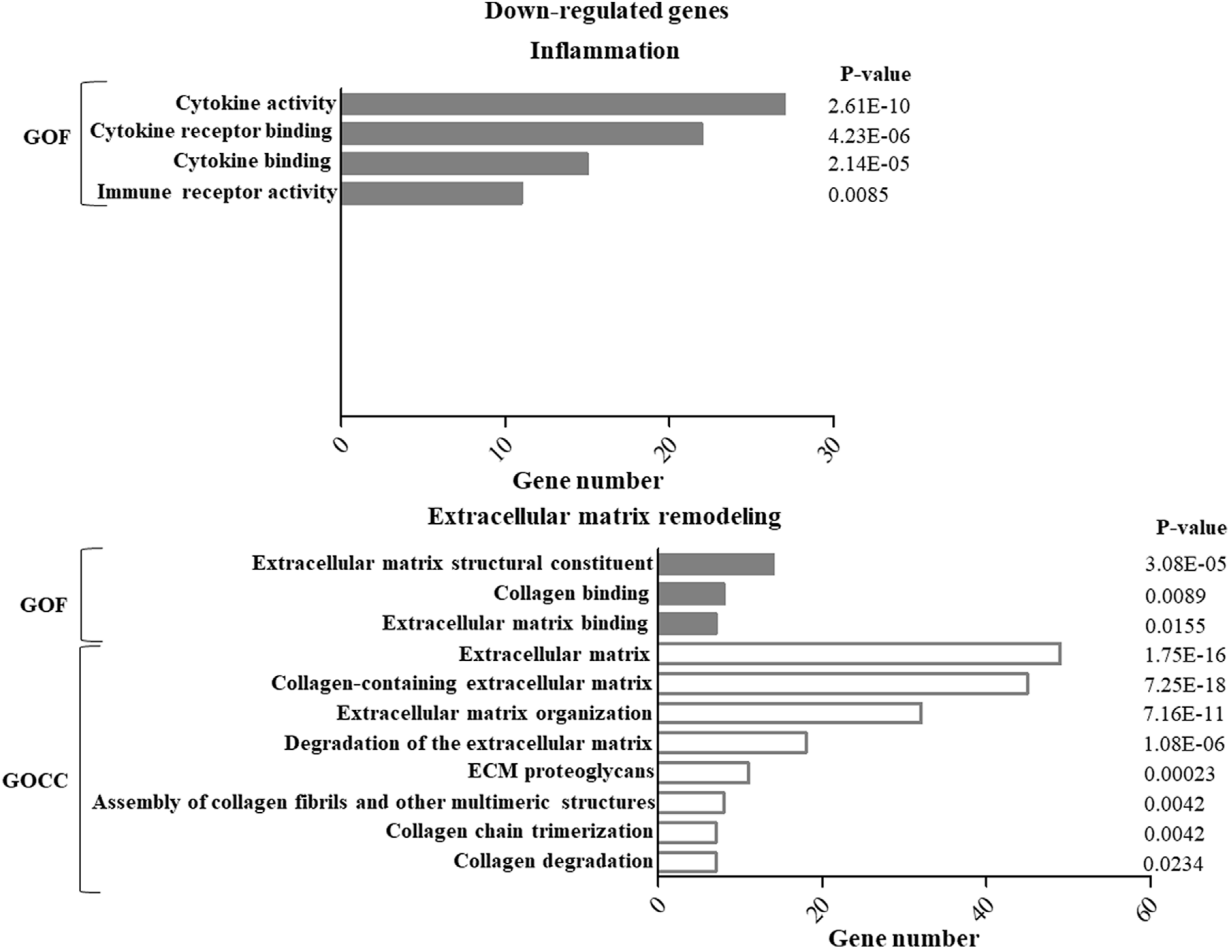
GO enrichment analysis of down-regulated genes in ASCs after IL-1β priming. Down-regulated genes were analyzed using the GO function (GOF) and cellular component (GOCC) collections. A GO term was significantly enriched if *p*-value ≤ 0.05 and FDR ≤0.05. Genes could be comprised in more than one term depending on the function of the encoded protein. The graph shows the most relevant GO terms. The GO term name is reported on the *y*-axis; the number of enriched genes for each term is indicated on the *x*-axis. GO terms are listed by decreasing number of genes. The *p* value for each GO term is indicated.

GO enrichment analysis of up- or down-regulated genes in ASCs after IL-1β priming allowed identifying specific functions and cellular components putatively modulated by this stimulation. In particular, for both the up- (Figure 1) and down- (Figure 2) regulated genes, specific pathways of interest in the OA context, involved in the modulation of inflammation and remodeling of the extracellular matrix, were identified. Both up-and down-regulated genes appeared to modulate the cytokine activity in the inflammatory context and the remodeling of collagen and proteoglycans. Up-regulated genes influenced also the chemokine activity.

**FIGURE 3:**
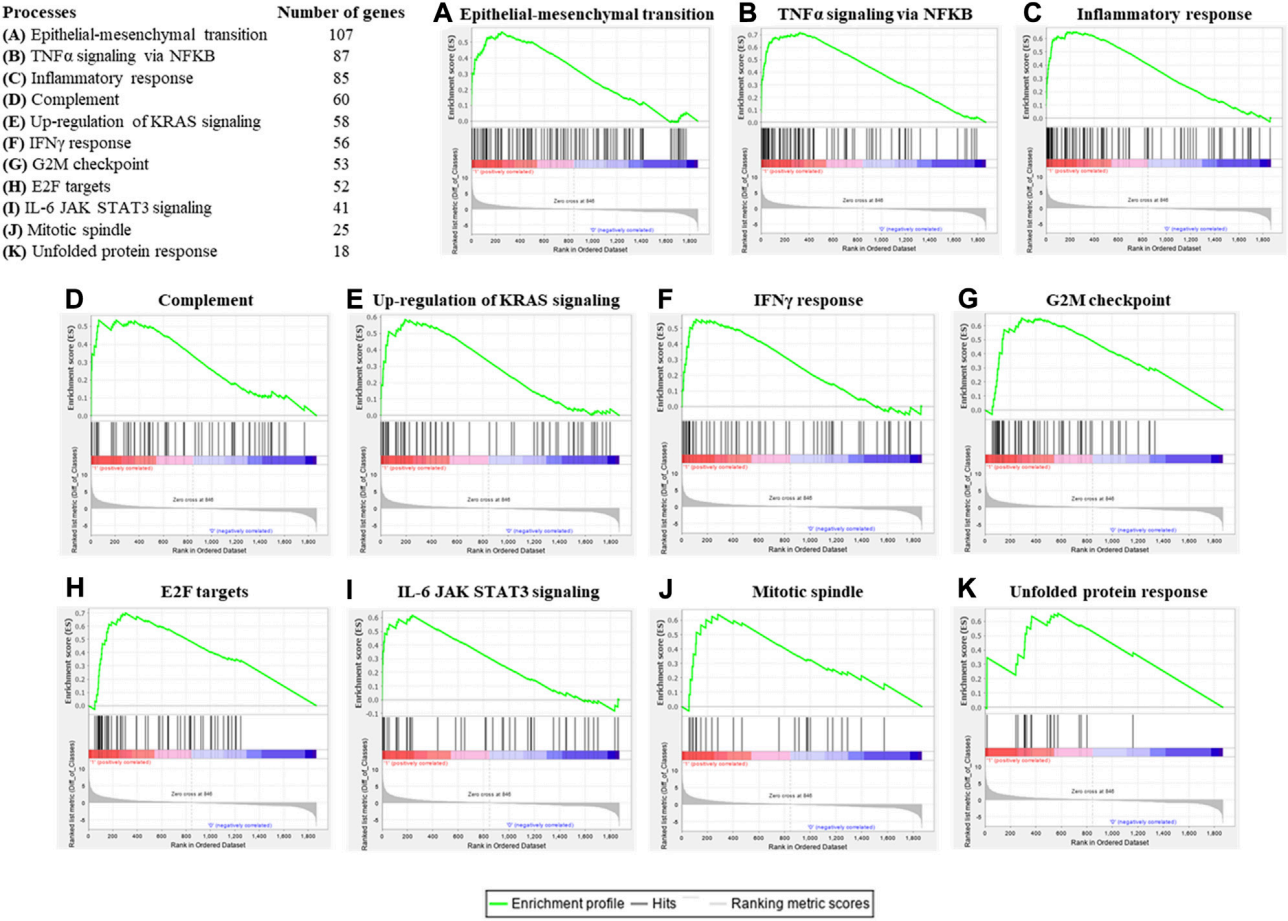
Enrichment plots of significant gene sets belonging to the **(H)** collection by GSEA. Table shows the hallmark processes identified by GSEA as significantly enriched (*p* value and q value < 0.05) and the number of genes belonging to each gene set and that was detectable also in the gene array. Enrichment plots for epithelial-mesenchymal transition **(A)**, TNFα signaling *via* NF-KB **(B)**, inflammatory response **(C)**, complement **(D)**, up-regulation of KRAS signaling **(E)**, IFNγ response **(F)**, G2M checkpoint **(G)**, E2F targets **(H)**, IL-6 JAK STAT3 signaling **(I)**, mitotic spindle **(J)** and unfolded protein response **(K)** were shown. An enrichment plot shows the gene set name (top), the running enrichment score (green curve), the positions of the gene set hits on the rank ordered list in GSEA (black bars), and the rank ordered list according to the signal-to-noise metric (bottom). Red indicates the up-regulation, whereas blue indicates the down-regulation of genes in the gene expression profile of ASCs after IL-1β priming.

To assess the concordant enrichment of functionally related genes in ASCs after IL-1β priming, we performed a GSEA between ASCs with or without IL-1β priming using Hallmark (H) as gene collection (Table 1). GSEA identified 11 significantly positively enriched gene sets in the inflamed sample (*p*-value<0.05 and q-value<0.05, Figure 3). The main processes enriched according to the GSEA are related to the inflammatory response (TNFα signaling *via* NF-KB, complement, IFNγ response, IL-6 JAK STAT3 signaling) and to the cell proliferative processes (epithelial-mesenchymal transition, up-regulation of KRAS signaling, G2M checkpoint, E2F targets, mitotic spindle, unfolded protein response). No gene sets were found to be significantly enriched in the non-primed sample.

**TABLE 1:** GSEA results comparing the gene expression profile between ASCs primed or not with IL-1β using H collection.

GENE SET NAME^a^	SIZE^b^	ES^c^	NES^d^	NOM p-val^e^	FDR q-val^f^
HALLMARK_TNFA_SIGNALING_*VIA*_NFKB	87	0.7174	2.4787	0.0000	0.0000
HALLMARK_INFLAMMATORY_RESPONSE	85	0.6483	2.1913	0.0000	0.0000
HALLMARK_E2F_TARGETS	52	0.6994	2.1870	0.0000	0.0000
HALLMARK_G2M_CHECKPOINT	53	0.6549	2.0292	0.0000	0.0007
HALLMARK_EPITHELIAL_MESENCHYMAL_TRANSITION	107	0.5648	2.0150	0.0000	0.0010
HALLMARK_KRAS_SIGNALING_UP	58	0.5856	1.8716	0.0000	0.0038
HALLMARK_IL6_JAK_STAT3_SIGNALING	41	0.6172	1.8416	0.0000	0.0051
HALLMARK_MITOTIC_SPINDLE	25	0.6410	1.7581	0.0038	0.0132
HALLMARK_INTERFERON_GAMMA_RESPONSE	56	0.5581	1.7542	0.0000	0.0123
HALLMARK_COMPLEMENT	60	0.5356	1.6985	0.0017	0.0185
HALLMARK_UNFOLDED_PROTEIN_RESPONSE	18	0.6561	1.6254	0.0175	0.0329
HALLMARK_COAGULATION	48	0.5041	1.5395	0.0269	0.0606
HALLMARK_ANGIOGENESIS	18	0.5819	1.4621	0.0709	0.1000
HALLMARK_ALLOGRAFT_REJECTION	93	0.4163	1.4598	0.0245	0.0942
HALLMARK_MYC_TARGETS_V1	27	0.5169	1.4098	0.0687	0.1267
HALLMARK_GLYCOLYSIS	48	0.4507	1.3847	0.0668	0.1406
HALLMARK_HYPOXIA	60	0.4120	1.3287	0.0874	0.1853
HALLMARK_MTORC1_SIGNALING	45	0.4351	1.3253	0.1164	0.1779
HALLMARK_ESTROGEN_RESPONSE_LATE	53	0.3920	1.2535	0.1503	0.2484
HALLMARK_ESTROGEN_RESPONSE_EARLY	47	0.4039	1.2469	0.1693	0.2440
HALLMARK_IL2_STAT5_SIGNALING	58	0.3518	1.1223	0.2669	0.4141
HALLMARK_P53_PATHWAY	53	0.3423	1.0734	0.3328	0.4796
HALLMARK_INTERFERON_ALPHA_RESPONSE	17	0.3847	0.9672	0.4695	0.6572
HALLMARK_APICAL_JUNCTION	58	0.2963	0.9478	0.5441	0.6705
HALLMARK_UV_RESPONSE_UP	48	0.2947	0.9251	0.5887	0.6902
HALLMARK_FATTY_ACID_METABOLISM	19	0.3220	0.8212	0.6992	0.8654
HALLMARK_XENOBIOTIC_METABOLISM	38	0.2704	0.7966	0.7580	0.8743
HALLMARK_SPERMATOGENESIS	16	0.2599	0.6462	0.9176	1.0000
HALLMARK_PI3K_AKT_MTOR_SIGNALING	38	0.1744	0.5166	0.9964	1.0000
HALLMARK_DNA_REPAIR	15	0.2199	0.5153	0.9902	0.9942

^a^ Gene sets belonging to the MSigDB, Hallmark collection. Enrichment analysis was performed with the GSEA, tool. Data have been unlogged prior to any analysis.

^b^ ES (enrichment score) indicates the maximum deviation from zero encountered in a random walk for a gene set.

^c^ NES (normalized enrichment score) indicates the fraction between the ES, and the mean of the ES, against 1,000 permutations of the gene sets. Gene sets are sorted by decreasing order of NES.

^d^ NOM *p*-value indicates the probability that an ES, equal to or higher than that found for the gene set may be observed by chance. Gene sets with a NOM *p*-value lower than 0.05 are considered significant.

^e^ FDR q-value is the estimated probability that the normalized enrichment score represents a false positive finding. Values ≤0.05 are considered acceptable.

### Modulation of T cell survival and phenotypes

Functional co-culture tests of ASCs with T cells and macrophages were performed to assess the immunomodulatory ability of primed ASCs predicted by the gene array analysis.

The co-culture of primed and non-primed ASCs and PBMCs induced a decrease in the survival rate of CD3^+^ T lymphocytes compared to control (*p* = 0.074 for primed ASCs and *p* = 0.012 for non-primed ASCs). This trend was not significantly different between primed and non-primed cells (*p* = 0.499).

The analysis of T cell phenotype showed a decrease in the percentage of CD4^+^ T cells in all the co-culture conditions, albeit this decrease was not significant both for primed and non-primed ASCs compared to the control condition represented by non-cocultured T-cells (*p* = 0.381 and *p* = 0.068, respectively). On the other hand, an increase in the percentage of CD8^+^ T cells was observed in all the co-culture conditions with no significant differences compared to the control condition (*p* = 0.148 for primed and *p* = 0.766 for non-primed ASCs). Finally, the potential to modulate the T cell phenotype was similar for both primed and non-primed cells (*p* = 0.147 for CD4^+^ T cell modulation and *p* = 0.541 for CD8^+^ T cells modulation) (Table 2).

**TABLE 2:** Modulation of T cells survival and phenotype by ASCs primed or not with IL-1β.

	PBMCs	ASCs	
		NT	+ IL-1β
CD3	91.0 ± 0.70	88.6 ± 0.65^**^	89.1 ± 1.15^^^
CD4	59.2 ± 1.27	56.8 ± 1.14^^^	58.3 ± 0.95
CD8	28.5 ± 0.62	28.8 ± 1.31	29.3 ± 0.46

Level of significance: **p = 0.01; ^tendency 0.05 < *p* ≤ 0.1 vs*.* PBMC., Values are presented as mean ± SD (*n* = 3). NT (not primed).

### IL-1β primed ASCs promote an anti-inflammatory macrophage polarization

**FIGURE 4:**
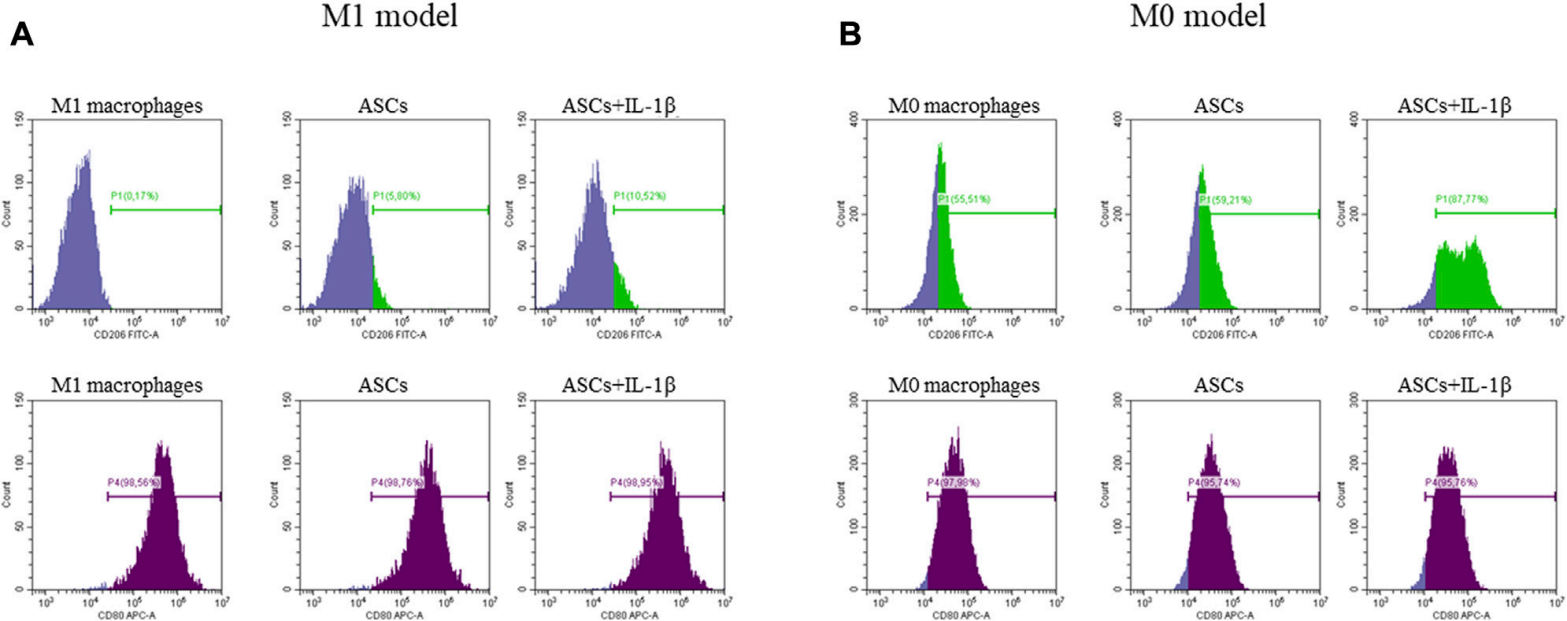
Expression of anti-inflammatory (CD206, green) and pro-inflammatory (CD80, purple) markers by M1 **(A)** and M0 **(B)** macrophages after co-culture with ASCs primed or non-primed with IL-1β. The percentage of positive cells is indicated on each single plot.

ASCs primed or non-primed with IL-1β and co-cultured with polarized M1 macrophages showed a potential to modify the CD80^+^/CD206^+^ ratio, promoting an increase of the CD206^+^ M2a anti-inflammatory marker in comparison with M1 macrophages alone. This effect was more evident in IL-1β primed ASCs (mean CD80^+^/CD206^+^ ratio: 49.0 ± 10.4; *p* = 0.006) than in non-primed cells (mean CD80^+^/CD206^+^ ratio: 61.7 ± 10.8; *p* = 0.04). Despite both primed and non-primed ASCs promoted an increase of the expression of the anti-inflammatory marker CD206, in this experimental set-up, including M1 macrophages, the co-culture with ASCs was not sufficient to induce a decrease of the expression of the pro-inflammatory marker CD80 (Figure 4A).

Similarly, when non-primed or primed ASCs were co-cultured with un-polarized M0 macrophages, the ability of ASCs to modify the CD80^+^/CD206^+^ ratio in favor of an anti-inflammatory macrophage phenotype was evident in both conditions, but more significantly in the primed cells (mean CD80^+^/CD206^+^ ratio: 1.6 ± 0.1, *p* = 0.001 for non-primed ASCs; and mean CD80^+^/CD206^+^ ratio: 0.7 ± 0.2, *p* = 0.0002 for primed ASCs). In this experimental set-up, a marked increase of CD206 expression was observed after co-culture of M0 macrophages with primed or non-primed ASCs, along with a slight decrease of the pro-inflammatory marker CD80 (Figure 4B).

## Discussion

The main finding of the present study is the ability of IL-1β to modulate in adipose-derived stem cells specific genes related to inflammation. Functional tests confirmed the enhanced immunomodulatory potential of the primed ASCs on macrophages. This potential is exploitable to preserve joint homeostasis and possibly ameliorate joint state in an OA context, in which M1 macrophages promote and sustain the inflammatory microenvironment through the interactions with synovial fibroblasts and chondrocytes (Xie et al., 2019). On the other hand, the IL-1β priming of ASCs did not enhance the slight immunomodulatory effect on T cells observed for non-primed ASCs, assessed as the ability to decrease CD3^+^ T cells survival and modulate the CD4^+^/CD8^+^ ratio. Our analysis showed that IL-1β is putatively able to activate TNFα signaling *via* NF-KB and cell response to IFNγ in ASCs. Previous studies have shown that TNFα triggers the immunosuppressive function of MSCs in inhibiting T cells proliferation (Dorronsoro et al., 2014). Similarly to TNFα, stimulation with IFNγ improves the ability of MSCs of suppressing the proliferation of CD4^+^ and CD8^+^ T lymphocytes (Krampera et al., 2006). Despite the activation of TNFα and IFNγ signaling, no enhancement of T cells modulation was observed after IL-1β priming in our study.

Based on these evidences, the general consideration that MSCs amplify their secretory activity following priming with inflammatory stimuli to counterbalance ongoing immune responses (de Witte et al., 2015) should be considered with more attention in the light of the specific inflammatory stimulus. ASCs regulate the immune system through two main mechanisms: a direct mechanism *via* cell-cell communication, and an indirect one, mediated by the secretion of soluble factors (Soleymaninejadian et al., 2012) and extracellular vesicles (de Witte et al., 2015). The most relevant immunomodulatory factors known to be highly secreted by MSCs include TGF-β and IL-6, the chemo-attractants IL-8, CCL2, CCL8, and prostaglandins E2 and F1 (Hoogduijn et al., 2010). MSCs also express ICAM-1 and VCAM-1, molecules inducible also by the concomitant presence of IFNγ and inflammatory cytokines (TNFα or IL-1β) (Ren et al., 2008). The expression of these adhesion molecules allows the recruitment of activated immune cells to close proximity of MSCs, thereby increasing the immune cell exposure to anti-inflammatory signals (Ren et al., 2008). In our study, we observed that IL-1β priming of ASCs was able to promote the up-regulation of several of these genes, such as IL-6, CCL2, CCL8, ICAM-1 and VCAM-1, thus sustaining the hypothesis that IL-1β enhances the possibility of ASCs to interact with immune cells and thus exert their immunomodulatory function. Among the main processes identified in the present study by gene ontology and gene sets enrichment analysis, the ones related to inflammatory responses, such as IL-6, TNFα and IFNγ signaling, are the most relevant in the OA context. In particular, IL-6 mediates both pro- and anti-inflammatory effects and modulates T cell response through classical signaling and trans-signaling (Scheller et al., 2011). The dual activity of IL-6 confers to this mediator a sort of homeostatic activity, possibly explaining the similar effect of primed and non-primed ASCs on T cells. Considering the other two mediators, the effects of IFNγ on MSCs have been studied most abundantly, but it was reported that also TNFα has potent effects on MSCs (de Witte et al., 2015). In particular, TNFα has similar, but less pronounced, effects on MSCs in terms of modulation of inflammatory mediators (English et al., 2007; Prasanna et al., 2010; Roemeling-van Rhijn et al., 2013). However, controversial findings have been reported. While some studies have shown that both TNFα and IFNγ priming trigger the immunosuppressive function of MSCs on T cells (Dorronsoro et al., 2014) (27), other studies have reported that the stimulation with either TNFα or IFNγ alone does not promote the immunosuppressive capacity of MSCs (English et al., 2007; Prasanna et al., 2010), but a combination of IFNγ and TNFα is needed (Ren et al., 2008). In our study, TNFα was up-regulated in primed ASCs, but the concomitant down-regulation of IFNγ likely accounts for the lack of a strong immunosuppressive effect of ASCs on T cells.

In our study, the more relevant functional confirmation of the immunomodulatory ability of IL-1β primed ASCs was the ability to influence macrophage polarization. Our macrophage polarization assay proved the enhanced ability of ASCs to modulate macrophage phenotype after priming with IL-1β. In particular, primed ASCs showed a stronger ability to shift un-polarized macrophages towards an anti-inflammatory phenotype, rather than to switch macrophages from M1 towards M2 phenotype. The observed up-regulation of IL-4 and IL-13, known as cytokines able to promote the development of anti-inflammatory M2 macrophages (Mosser and Edwards, 2008; Sica and Mantovani, 2012), was in line with this anti-inflammatory induction. A previous study has also investigated the interactions between ASCs and macrophages (Ortiz-Virumbrales et al., 2020), showing results in complete agreement with our data, with the greatest modulatory effect of ASCs observed on M0 un-polarized macrophages through the induction of an anti-inflammatory profile. In our experiments, the co-culture of ASCs and macrophages resulted in a greater increase in CD206 surface expression, whereas the expression of CD80 was not altered both in macrophages during M1 polarization and in M0 un-polarized macrophages.

These preliminary data are particular relevant considering a possible translation of this priming strategy of the ASCs into the clinical practice. Considering the injective therapies ASC-based to treat OA, it was previously suggested that an inflamed milieu was able to prime the injected cells, making them able to exert their homeostatic function at best (Lopa et al., 2019).

The main limitation of this study is related to the limited number of donors from which ASCs were derived. This does not allow generalizing these results without further research, but the approach to validate with functional tests the predictions provided by “omics” proved to be useful to identify macrophages as the subset of immune cells more responsive in terms of immunomodulation to IL-1β primed ASCs.

In conclusion, overwhelming evidence ascribes inflammatory processes a pivotal role in OA onset and progression as well as in the generation of OA symptoms. Thus, the use of ASCs for OA treatment and pathology resolution is of great interest and a major goal to achieve in the coming years. This interest resides in their ability to be easily isolated and expanded, in their multi-potential differentiation properties (Mohamed-Ahmed et al., 2018) and above all in their immunosuppressive potential (Ceccarelli et al., 2020). However, it has been previously demonstrated that the therapeutic ability of ASCs highly dependent on the cytokine microenvironment which they are exposed to (Bernardo and Fibbe, 2013; Sayegh et al., 2019). Priming of stem cells with bioactive molecules, among them IL-1β, is hence proposed as a way to foster the therapeutic potential of these cells (Fan et al., 2012; Jauković et al., 2020).

The present study identified IL-1β priming as useful to enhance the ability of ASCs to modulate macrophages toward an anti-inflammatory phenotype. This is important especially in the OA context where normalizing the aberrant M1/M2 ratio has been suggested as an effective strategy to counteract the inflammatory component of the pathology and supports the idea of tailoring ASCs immunomodulatory potential to enhance their therapeutic efficacy. Future *in vivo* studies are needed to confirm this data and to provide the basis for the translation of this approach into the clinical practice.

## Data Availability

The datasets presented in this study can be found in online repositories. The names of the repository/repositories and accession number(s) can be found below: https://osf.io/ymp3g/?view_only=5f5396ab065e4666b56c6841c077c475.
