# Supplementary material for: Inflammatory priming with IL-1β promotes the immunomodulatory behavior of adipose derived stem cells
Source: Front Bioeng Biotechnol. 2022 Oct 20;10:1000879. doi: 10.3389/fbioe.2022.1000879 (PMC9632288; doi:10.3389/fbioe.2022.1000879)
Supplement: Supplementary file 1 [file Table1.DOCX]

**Supplementary Table 1.** Genes up- and down-regulated by the IL-1β priming in the ASCs.

| **Up-regulated genes** | | **Down-regulated genes** | |
| --- | --- | --- | --- |
| **Gene symbol** | **Fold change** | **Gene symbol** | **Fold change** |
| *CXCL6* | 4251.5 | *ADH1B* | 0.01 |
| *CXCL8* | 528.4 | *FOXG1* | 0.02 |
| *CSF2* | 514.8 | *CD28* | 0.02 |
| *CXCL1* | 365.2 | *C9orf135* | 0.03 |
| *CCL5* | 358.3 | *SLC40A1* | 0.03 |
| *CSF3* | 166.9 | *CHRNA3* | 0.03 |
| *CXCL5* | 157.8 | *FAM189A2* | 0.03 |
| *CCL20* | 110.0 | *CD40LG* | 0.03 |
| *CCL11* | 101.0 | *ANGPT2* | 0.04 |
| *MMP3* | 80.3 | *SCARA5* | 0.04 |
| *CXCL3* | 77.0 | *ACTC1* | 0.04 |
| *MMP12* | 62.0 | *IFNG* | 0.05 |
| *CCL7* | 36.2 | *SMOC2* | 0.05 |
| *ELF3* | 33.6 | *FSD1* | 0.1 |
| *STC1* | 30.3 | *IGFBP5* | 0.1 |
| *MMP10* | 26.1 | *ADAMTS15* | 0.1 |
| *CCL2* | 24.1 | *LPL* | 0.1 |
| *IL11* | 21.2 | *IL12RB1* | 0.1 |
| *SAA2* | 19.8 | *ASPN* | 0.1 |
| *MMP1* | 19.4 | *SLCO1B1* | 0.1 |
| *UBD* | 19.2 | *KRT18* | 0.1 |
| *LRRC15* | 18.4 | *VCX3A* | 0.1 |
| *CA9* | 17.6 | *VEGFD* | 0.1 |
| *LIF* | 16.8 | *SELENOP* | 0.1 |
| *IL1B* | 16.2 | *SOSTDC1* | 0.1 |
| *TDO2* | 14.6 | *ADRA2B* | 0.1 |
| *IL24* | 14.1 | *SOX10* | 0.1 |
| *TFPI2* | 12.9 | *NEURL3* | 0.1 |
| *PTPRE* | 12.5 | *CCN3* | 0.1 |
| *IDO1* | 12.1 | *TNFSF10* | 0.1 |
| *CCL19* | 12.0 | *COX6A2* | 0.1 |
| *DNER* | 11.9 | *GNG4* | 0.1 |
| *AMIGO2* | 11.9 | *KCNMB4* | 0.1 |
| *IL23A* | 11.5 | *TPSAB1* | 0.1 |
| *CCL13* | 10.3 | *TNNI2* | 0.1 |
| *CCL8* | 10.2 | *IL25* | 0.1 |
| *PRL* | 10.2 | *FABP6* | 0.1 |
| *NEFM* | 9.5 | *CHGA* | 0.1 |
| *CCL3* | 9.2 | *VCX2* | 0.1 |
| *CXCL2* | 9.1 | *HGF* | 0.1 |
| *TMEM132A* | 8.7 | *DLGAP1* | 0.1 |
| *TNFAIP6* | 8.6 | *CCR5* | 0.1 |
| *ASCL2* | 8.5 | *PALD1* | 0.1 |
| *NRXN3* | 8.4 | *FGFBP1* | 0.1 |
| *GIMAP8* | 8.0 | *SHISA2* | 0.1 |
| *MMP9* | 8.0 | *CA14* | 0.1 |
| *LRIG1* | 7.8 | *KRT19* | 0.1 |
| *SLC22A3* | 7.3 | *SCIN* | 0.1 |
| *TSPAN13* | 7.3 | *PPL* | 0.1 |
| *SLC2A1* | 7.2 | *AMBP* | 0.1 |
| *MYOCD* | 7.0 | *CUX2* | 0.1 |
| *CXCL10* | 6.9 | *C1orf100* | 0.1 |
| *HBEGF* | 6.9 | *ACAN* | 0.1 |
| *IL1RN* | 6.8 | *CYP4F8* | 0.1 |
| *LTB* | 6.8 | *CCL22* | 0.1 |
| *IFI44L* | 6.8 | *MAP1LC3C* | 0.1 |
| *PLAAT2* | 6.6 | *BMP4* | 0.1 |
| *SLC7A2* | 6.5 | *PDGFD* | 0.1 |
| *EPHB2* | 6.4 | *HEY2* | 0.1 |
| *TRIM36* | 6.3 | *PLAC8* | 0.1 |
| *CYP2F1* | 6.3 | *ADIPOQ* | 0.1 |
| *PTGS2* | 5.9 | *NOX1* | 0.1 |
| *MMP13* | 5.9 | *ITGA9* | 0.1 |
| *BUB1* | 5.8 | *VIT* | 0.1 |
| *DEPDC1B* | 5.8 | *IL10* | 0.1 |
| *PCDHB11* | 5.8 | *A2M* | 0.1 |
| *CDC25C* | 5.6 | *ERC2* | 0.2 |
| *NKAIN1* | 5.6 | *TOX* | 0.2 |
| *RASGRP1* | 5.5 | *ADRA2C* | 0.2 |
| *SGO1* | 5.5 | *ERBB3* | 0.2 |
| *SPC25* | 5.4 | *NTF4* | 0.2 |
| *IL6* | 5.3 | *OTULINL* | 0.2 |
| *SELP* | 5.3 | *ADCY1* | 0.2 |
| *DACT1* | 5.2 | *FGF18* | 0.2 |
| *IL4* | 5.2 | *NGEF* | 0.2 |
| *IL1A* | 5.1 | *KRT14* | 0.2 |
| *ERCC6L* | 5.1 | *CLEC3B* | 0.2 |
| *ANKRD1* | 5.1 | *SPTB* | 0.2 |
| *NRP2* | 5.1 | *RETN* | 0.2 |
| *IL32* | 5.0 | *OMD* | 0.2 |
| *CCR6* | 5.0 | *PRELP* | 0.2 |
| *GPR160* | 5.0 | *CDH1* | 0.2 |
| *CCDC181* | 4.9 | *ACSBG1* | 0.2 |
| *PLG* | 4.9 | *LDB2* | 0.2 |
| *ASGR1* | 4.8 | *SCNN1A* | 0.2 |
| *CDC20* | 4.8 | *BSPRY* | 0.2 |
| *RLN2* | 4.8 | *ANXA3* | 0.2 |
| *ATP1A2* | 4.8 | *NPR1* | 0.2 |
| *KYNU* | 4.8 | *CCL23* | 0.2 |
| *SERPINE1* | 4.7 | *CTNND2* | 0.2 |
| *E2F1* | 4.6 | *SYT4* | 0.2 |
| *SOD2* | 4.6 | *CTLA4* | 0.2 |
| *WWC1* | 4.6 | *IL22* | 0.2 |
| *IL17C* | 4.6 | *LSP1* | 0.2 |
| *LYVE1* | 4.5 | *COL2A1* | 0.2 |
| *MAGEL2* | 4.5 | *PRDM13* | 0.2 |
| *EHF* | 4.5 | *IL17RD* | 0.2 |
| *LTBP1* | 4.5 | *CD86* | 0.2 |
| *LMNB1* | 4.5 | *RLN1* | 0.2 |
| *PTAFR* | 4.4 | *AQP1* | 0.2 |
| *NAMPT* | 4.4 | *TUBB1* | 0.2 |
| *C1QTNF1* | 4.4 | *EFNA1* | 0.2 |
| *ANLN* | 4.4 | *GLS2* | 0.2 |
| *CHD7* | 4.4 | *SLC15A1* | 0.2 |
| *CD163* | 4.4 | *MAP2K6* | 0.2 |
| *PCYT1B* | 4.3 | *PPP2R1B* | 0.2 |
| *DLGAP5* | 4.3 | *JAG1* | 0.2 |
| *BACE2* | 4.3 | *C4orf19* | 0.2 |
| *ADGRB1* | 4.2 | *ADAMTS5* | 0.2 |
| *CXCL9* | 4.2 | *PDE6B* | 0.2 |
| *AUNIP* | 4.2 | *ADAMTS8* | 0.2 |
| *FANCA* | 4.1 | *NODAL* | 0.2 |
| *RASL11B* | 4.1 | *ALAS2* | 0.2 |
| *CCL26* | 4.1 | *S1PR1* | 0.2 |
| *BUB1B* | 4.0 | *IL5* | 0.2 |
| *SOX10* | 4.0 | *GDF5* | 0.2 |
| *LPAR4* | 4.0 | *PTH1R* | 0.2 |
| *PCSK1* | 3.9 | *LRRN4CL* | 0.2 |
| *FGF2* | 3.9 | *H2AC4* | 0.2 |
| *TNF* | 3.9 | *NPTX2* | 0.2 |
| *TPH1* | 3.9 | *MYL4* | 0.2 |
| *HMMR* | 3.9 | *KHK* | 0.2 |
| *SLC16A3* | 3.8 | *PACSIN1* | 0.2 |
| *MKI67* | 3.8 | *ZNF676* | 0.2 |
| *WNK3* | 3.8 | *RSPO3* | 0.2 |
| *DTL* | 3.8 | *CCR1* | 0.2 |
| *OIP5* | 3.8 | *EPHB6* | 0.2 |
| *ARHGAP25* | 3.8 | *COL14A1* | 0.2 |
| *FST* | 3.8 | *OGN* | 0.2 |
| *TP63* | 3.8 | *SMPDL3B* | 0.2 |
| *NPPC* | 3.8 | *SCT* | 0.2 |
| *NOD2* | 3.8 | *MGP* | 0.2 |
| *RLN1* | 3.7 | *NR3C2* | 0.2 |
| *MYEF2* | 3.7 | *CCR3* | 0.2 |
| *BLM* | 3.7 | *CKMT1B* | 0.2 |
| *ICAM1* | 3.7 | *TDRKH* | 0.2 |
| *GRM8* | 3.7 | *CTSV* | 0.2 |
| *KIF20A* | 3.7 | *CDKN1C* | 0.2 |
| *ADAMTS4* | 3.7 | *KCNB1* | 0.2 |
| *TOP2A* | 3.6 | *OR2A7* | 0.2 |
| *LMO2* | 3.6 | *ANGPTL1* | 0.2 |
| *PLCB4* | 3.6 | *COL9A2* | 0.2 |
| *MXRA7* | 3.6 | *C5AR1* | 0.2 |
| *MYC* | 3.6 | *SYT1* | 0.2 |
| *TACC3* | 3.6 | *DPYS* | 0.2 |
| *HEYL* | 3.5 | *TNNT2* | 0.2 |
| *PLK4* | 3.5 | *IFNA4* | 0.2 |
| *PIMREG* | 3.5 | *C3AR1* | 0.2 |
| *GABRB3* | 3.5 | *MFAP4* | 0.2 |
| *PBK* | 3.5 | *RNF130* | 0.2 |
| *COL9A1* | 3.5 | *HHIP* | 0.2 |
| *LRRN3* | 3.5 | *SLC25A4* | 0.2 |
| *PMEPA1* | 3.4 | *CHDH* | 0.2 |
| *HTR7* | 3.4 | *ARHGAP26* | 0.2 |
| *IL19* | 3.4 | *PDE5A* | 0.2 |
| *STEAP1* | 3.4 | *ADGRG2* | 0.2 |
| *PIM2* | 3.4 | *SYT13* | 0.3 |
| *SOX3* | 3.4 | *KIT* | 0.3 |
| *NEFL* | 3.4 | *ARAP2* | 0.3 |
| *PLK1* | 3.4 | *SOX8* | 0.3 |
| *SOX11* | 3.4 | *PADI2* | 0.3 |
| *ITGA1* | 3.4 | *HS3ST2* | 0.3 |
| *KIF1A* | 3.4 | *PLG* | 0.3 |
| *EBI3* | 3.4 | *ABCG1* | 0.3 |
| *CCNA2* | 3.4 | *NLGN3* | 0.3 |
| *IL12B* | 3.3 | *ROR1* | 0.3 |
| *GDNF* | 3.3 | *DSC2* | 0.3 |
| *WNT5A* | 3.3 | *TMEM30B* | 0.3 |
| *MYB* | 3.2 | *STMN3* | 0.3 |
| *PDE3B* | 3.2 | *RIMS3* | 0.3 |
| *CLIC6* | 3.2 | *SGCG* | 0.3 |
| *IGF2BP1* | 3.2 | *PRDM14* | 0.3 |
| *IL17B* | 3.2 | *ESR1* | 0.3 |
| *CDH2* | 3.2 | *GABRA5* | 0.3 |
| *PTBP2* | 3.2 | *MS4A1* | 0.3 |
| *NRG1* | 3.2 | *CHRM2* | 0.3 |
| *FZD3* | 3.2 | *DAPK1* | 0.3 |
| *PCDHB12* | 3.2 | *GHR* | 0.3 |
| *GALNT17* | 3.1 | *VWF* | 0.3 |
| *PAK6* | 3.1 | *RPRM* | 0.3 |
| *CCNB1* | 3.1 | *ST14* | 0.3 |
| *BMPER* | 3.1 | *PDE9A* | 0.3 |
| *YPEL1* | 3.1 | *EPYC* | 0.3 |
| *FABP4* | 3.1 | *IL9* | 0.3 |
| *JPH3* | 3.1 | *RHOJ* | 0.3 |
| *F12* | 3.1 | *SNED1* | 0.3 |
| *CHST6* | 3.1 | *IL15* | 0.3 |
| *VRTN* | 3.1 | *SLC14A1* | 0.3 |
| *CD38* | 3.1 | *RASL12* | 0.3 |
| *RAP1GAP2* | 3.1 | *SOX11* | 0.3 |
| *CD37* | 3.1 | *NECAB1* | 0.3 |
| *KCNS3* | 3.1 | *PRKCB* | 0.3 |
| *CDC6* | 3.0 | *IL1R2* | 0.3 |
| *TGFB2* | 3.0 | *EXPH5* | 0.3 |
| *UBASH3B* | 3.0 | *TNFSF13B* | 0.3 |
| *GNG11* | 3.0 | *AGTR1* | 0.3 |
| *KCNE5* | 3.0 | *CHST8* | 0.3 |
| *EXO1* | 3.0 | *TNNI3* | 0.3 |
| *MAT1A* | 3.0 | *MZB1* | 0.3 |
| *GINS2* | 3.0 | *TGFB1* | 0.3 |
| *AREG* | 3.0 | *BMP5* | 0.3 |
| *GCH1* | 3.0 | *VAT1L* | 0.3 |
| *VEGFC* | 3.0 | *NAALAD2* | 0.3 |
| *CDH15* | 3.0 | *RSAD2* | 0.3 |
| *TLR2* | 3.0 | *ANKH* | 0.3 |
| *PFKFB3* | 3.0 | *MYO7A* | 0.3 |
| *VAV3* | 2.9 | *COMP* | 0.3 |
| *SALL4* | 2.9 | *AHSG* | 0.3 |
| *PFN3* | 2.9 | *NOG* | 0.3 |
| *ARHGEF16* | 2.9 | *AQP2* | 0.3 |
| *CELSR2* | 2.9 | *CDH5* | 0.3 |
| *MMP7* | 2.9 | *CDS1* | 0.3 |
| *IL36G* | 2.9 | *MAB21L2* | 0.3 |
| *RAD51AP1* | 2.9 | *ATP1A2* | 0.3 |
| *KLRB1* | 2.9 | *FGFR3* | 0.3 |
| *NFE2L3* | 2.9 | *TNNC2* | 0.3 |
| *MASP2* | 2.9 | *ADGRG6* | 0.3 |
| *CLDN3* | 2.8 | *SELE* | 0.3 |
| *ADRA2A* | 2.8 | *IGSF9* | 0.3 |
| *CACNA1H* | 2.8 | *PLPP3* | 0.3 |
| *CENPE* | 2.8 | *SCRG1* | 0.3 |
| *PAX8* | 2.8 | *FAT3* | 0.3 |
| *PDPN* | 2.8 | *MYBPH* | 0.3 |
| *TFR2* | 2.8 | *CD7* | 0.3 |
| *ITGA2* | 2.8 | *SNX10* | 0.3 |
| *PDLIM7* | 2.8 | *APOC1* | 0.3 |
| *IL17A* | 2.8 | *TXNIP* | 0.3 |
| *SLC35F1* | 2.8 | *CXADR* | 0.3 |
| *EPCAM* | 2.8 | *IGF1* | 0.3 |
| *SCT* | 2.8 | *IL16* | 0.3 |
| *CDC25A* | 2.8 | *CRYAB* | 0.3 |
| *PDE4D* | 2.8 | *BRINP1* | 0.4 |
| *OSM* | 2.8 | *PMEL* | 0.4 |
| *UGT1A8* | 2.8 | *CAMK2N1* | 0.4 |
| *INAVA* | 2.7 | *AMOT* | 0.4 |
| *SPINK5* | 2.7 | *DCN* | 0.4 |
| *FAM184A* | 2.7 | *RHOH* | 0.4 |
| *LY96* | 2.7 | *SFRP1* | 0.4 |
| *ACTA1* | 2.7 | *MPPED2* | 0.4 |
| *ESRP1* | 2.7 | *IGF2* | 0.4 |
| *CXCR3* | 2.7 | *COL5A3* | 0.4 |
| *EPB41L4B* | 2.7 | *GULP1* | 0.4 |
| *GFPT2* | 2.7 | *TGFB3* | 0.4 |
| *IGFBP7* | 2.7 | *BCAM* | 0.4 |
| *VSIG4* | 2.6 | *ACACB* | 0.4 |
| *FOXA3* | 2.6 | *SERPINB4* | 0.4 |
| *TMSB15A* | 2.6 | *STOM* | 0.4 |
| *NFKBIA* | 2.6 | *OGDHL* | 0.4 |
| *PFN1* | 2.6 | *PODXL* | 0.4 |
| *ATAD2* | 2.6 | *KDR* | 0.4 |
| *IL12A* | 2.6 | *NDUFA5* | 0.4 |
| *GABRB1* | 2.6 | *DHDH* | 0.4 |
| *PARM1* | 2.6 | *ANXA4* | 0.4 |
| *FXYD6* | 2.6 | *FAM149A* | 0.4 |
| *GDF9* | 2.6 | *CD69* | 0.4 |
| *CENPU* | 2.6 | *CIB2* | 0.4 |
| *ANGPT1* | 2.6 | *CD244* | 0.4 |
| *S100A8* | 2.6 | *LRRC8B* | 0.4 |
| *SERPINB2* | 2.6 | *CRHR1* | 0.4 |
| *TNFRSF8* | 2.5 | *THBS3* | 0.4 |
| *CNTN2* | 2.5 | *FGF6* | 0.4 |
| *PERP* | 2.5 | *L1TD1* | 0.4 |
| *PGF* | 2.5 | *RARA* | 0.4 |
| *PENK* | 2.5 | *GSC* | 0.4 |
| *RASL12* | 2.5 | *FCER1A* | 0.4 |
| *THBS1* | 2.5 | *PTCH1* | 0.4 |
| *AKR1B1* | 2.5 | *CD3G* | 0.4 |
| *ITGB3* | 2.5 | *CELSR1* | 0.4 |
| *CD79A* | 2.5 | *COL9A3* | 0.4 |
| *PCDH19* | 2.5 | *CILP2* | 0.4 |
| *H4C4* | 2.5 | *EFEMP1* | 0.4 |
| *CHAD* | 2.5 | *CD72* | 0.4 |
| *GDAP1L1* | 2.5 | *CCL21* | 0.4 |
| *S100A9* | 2.5 | *PROK2* | 0.4 |
| *CCL15* | 2.5 | *ADD2* | 0.4 |
| *IGFBP1* | 2.5 | *TEK* | 0.4 |
| *NAP1L2* | 2.4 | *COL11A2* | 0.4 |
| *PRODH* | 2.4 | *CASQ1* | 0.4 |
| *GPR27* | 2.4 | *TLR1* | 0.4 |
| *ALPK3* | 2.4 | *TMCC3* | 0.4 |
| *INHBA* | 2.4 | *IL6R* | 0.4 |
| *HELLS* | 2.4 | *TGFBR3* | 0.4 |
| *PRKDC* | 2.4 | *RAB6B* | 0.4 |
| *COL10A1* | 2.4 | *DSG2* | 0.4 |
| *HK2* | 2.4 | *RGMA* | 0.4 |
| *ZNF711* | 2.4 | *SYNE2* | 0.4 |
| *SDC4* | 2.4 | *INPP5D* | 0.4 |
| *ADIPOQ* | 2.4 | *MRPL2* | 0.4 |
| *ABL1* | 2.4 | *CLCA2* | 0.4 |
| *SLC7A7* | 2.4 | *FZD8* | 0.4 |
| *RPS6KA1* | 2.4 | *BCL2L13* | 0.4 |
| *NR4A2* | 2.4 | *GPD1* | 0.4 |
| *CDK2* | 2.4 | *FSTL4* | 0.4 |
| *GALNT3* | 2.4 | *RNF125* | 0.4 |
| *IL18* | 2.4 | *DAAM2* | 0.4 |
| *PPBP* | 2.3 | *PTGIS* | 0.4 |
| *RAD51* | 2.3 | *F11R* | 0.4 |
| *PURG* | 2.3 | *AHNAK* | 0.4 |
| *SLC35F2* | 2.3 | *SH2D3C* | 0.4 |
| *IL13* | 2.3 | *ZEB1* | 0.4 |
| *FANCD2* | 2.3 | *MYL1* | 0.4 |
| *SUCO* | 2.3 | *SHROOM2* | 0.4 |
| *TNFRSF12A* | 2.3 | *SNTG2* | 0.4 |
| *VCAM1* | 2.3 | *COL15A1* | 0.4 |
| *SEMA6A* | 2.3 | *WNT11* | 0.4 |
| *RETN* | 2.3 | *RIPOR2* | 0.4 |
| *ESPL1* | 2.3 | *EPO* | 0.4 |
| *BATF3* | 2.3 | *OCLN* | 0.4 |
| *EME1* | 2.3 | *SOHLH2* | 0.4 |
| *BRCA2* | 2.3 | *REEP1* | 0.4 |
| *CANX* | 2.3 | *SEMA3F* | 0.4 |
| *EREG* | 2.3 | *PHYHIP* | 0.4 |
| *SOX4* | 2.3 | *PDGFRB* | 0.4 |
| *MCM5* | 2.3 | *DCLK1* | 0.4 |
| *GDF6* | 2.3 | *KCND2* | 0.4 |
| *CHP2* | 2.3 | *CES1* | 0.4 |
| *ALOX15* | 2.3 | *TCL1A* | 0.4 |
| *KLK3* | 2.3 | *FZD5* | 0.4 |
| *C3* | 2.2 | *RALGPS1* | 0.4 |
| *TBX3* | 2.2 | *PTK2B* | 0.4 |
| *DRD4* | 2.2 | *APOL6* | 0.4 |
| *CFLAR* | 2.2 | *CD5* | 0.4 |
| *LDHA* | 2.2 | *SDC1* | 0.5 |
| *RASD1* | 2.2 | *F2RL1* | 0.5 |
| *GPRC5B* | 2.2 | *SLC39A10* | 0.5 |
| *CLGN* | 2.2 | *PAX1* | 0.5 |
| *RAMP3* | 2.2 | *AIM2* | 0.5 |
| *CLDN11* | 2.2 | *S100A10* | 0.5 |
| *IL17F* | 2.2 | *IMPA2* | 0.5 |
| *ONECUT2* | 2.2 | *SOD3* | 0.5 |
| *NETO2* | 2.2 | *NOS1AP* | 0.5 |
| *COL8A1* | 2.2 | *RAI2* | 0.5 |
| *STOX2* | 2.2 | *RAP1A* | 0.5 |
| *CXCL11* | 2.2 | *MSTN* | 0.5 |
| *ZNF253* | 2.2 | *ATP5PB* | 0.5 |
| *PMAIP1* | 2.2 | *IDH1* | 0.5 |
| *TNFRSF1B* | 2.2 | *SLCO4A1* | 0.5 |
| *CA12* | 2.2 | *PLA2G3* | 0.5 |
| *SERPINC1* | 2.2 | *RNF128* | 0.5 |
| *SELE* | 2.2 | *CD24* | 0.5 |
| *COL3A1* | 2.2 | *HLA-DRA* | 0.5 |
| *LOXL2* | 2.2 | *FCGR1A* | 0.5 |
| *NLGN4X* | 2.2 | *SLC29A2* | 0.5 |
| *AVPR2* | 2.2 | *PRRX1* | 0.5 |
| *SOX9* | 2.2 | *EPHX1* | 0.5 |
| *GPAM* | 2.2 | *ATP2B4* | 0.5 |
| *ANPEP* | 2.1 | *ITGBL1* | 0.5 |
| *MCAM* | 2.1 | *NRXN3* | 0.5 |
| *NTF3* | 2.1 | *PCDH7* | 0.5 |
| *RHO* | 2.1 | *PERP* | 0.5 |
| *IL21* | 2.1 | *MYLIP* | 0.5 |
| *CSF1R* | 2.1 | *NQO1* | 0.5 |
| *GLDC* | 2.1 | *IGFBP6* | 0.5 |
| *FTH1* | 2.1 | *BCL2* | 0.5 |
| *ETS2* | 2.1 | *ACVR2A* | 0.5 |
| *TNFAIP8* | 2.1 | *PARD6A* | 0.5 |
| *VEGFA* | 2.1 | *TWIST1* | 0.5 |
| *ETS1* | 2.1 | *ANAPC10* | 0.5 |
| *NCAM1* | 2.1 | *CLEC1A* | 0.5 |
| *SMPDL3B* | 2.1 | *CDKN2C* | 0.5 |
| *HTR1D* | 2.1 | *IL7* | 0.5 |
| *RUNX2* | 2.1 | *ACVR2B* | 0.5 |
| *BTG1* | 2.1 | *MFAP5* | 0.5 |
| *ZIK1* | 2.1 | *LEP* | 0.5 |
| *CXCR4* | 2.1 | *CHI3L1* | 0.5 |
| *CCL4* | 2.1 | *SEMA5A* | 0.5 |
| *TIMP1* | 2.0 | *ST6GAL1* | 0.5 |
| *LAT2* | 2.0 | *HTRA1* | 0.5 |
| *ELN* | 2.0 | *STOX2* | 0.5 |
| *UGT1A6* | 2.0 | *SLC44A1* | 0.5 |
| *SPDL1* | 2.0 | *ANXA11* | 0.5 |
| *SLC7A1* | 2.0 | *SPP1* | 0.5 |
| *STRBP* | 2.0 |  |  |
| *BRCA1* | 2.0 |  |  |
| *GPR19* | 2.0 |  |  |
| *BID* | 2.0 |  |  |
| *MPL* | 2.0 |  |  |
| *E2F3* | 2.0 |  |  |
| *PMEL* | 2.0 |  |  |
| *CXCL13* | 2.0 |  |  |
| *NFKB1* | 2.0 |  |  |
| *MYOD1* | 2.0 |  |  |
| *ASB1* | 2.0 |  |  |
| *CD209* | 2.0 |  |  |
